# The influence of midlife morbidity clusters on dementia risk: The ARIC study

**DOI:** 10.1002/alz.71110

**Published:** 2026-02-09

**Authors:** Elise Kinyanjui, Renee C. Groechel, Valerie Morrill, Keenan A. Walker, Anna M. Kucharska‐Newton, Thomas H. Mosley, Silvia Koton, David S. Knopman, Jordan Weiss, Rebecca F. Gottesman, Marco Egle

**Affiliations:** ^1^ Department of Neuroscience St. Mary's College of Maryland St. Mary's City Maryland USA; ^2^ National Institute of Neurological Disorders and Stroke Intramural Research Program, National Institutes of Health Bethesda Maryland USA; ^3^ Laboratory of Behavioral Neuroscience National Institute on Aging Intramural Research Program Baltimore Maryland USA; ^4^ Department of Epidemiology University of North Carolina at Chapel Hill Gillings School of Global Public Health Chapel Hill North Carolina USA; ^5^ Department of Medicine University of Mississippi Medical Center Jackson Mississippi USA; ^6^ Department of Nursing The Stanley Steyer School of Health Professions Tel Aviv University Tel Aviv Israel; ^7^ Department of Epidemiology Johns Hopkins University School of Public Health Baltimore Maryland USA; ^8^ Optimal Aging Institute and Department of Medicine New York University Grossman School of Medicine New York New York USA; ^9^ Department of Neurology Mayo Clinic College of Medicine and Science Rochester Minnesota USA

**Keywords:** Dementia, mortality, stroke, vascular risk factors

## Abstract

**INTRODUCTION:**

Understanding comorbidities’ combined impacts on dementia risk may offer a more comprehensive understanding of individuals’ risk. Using machine‐learning, we grouped individuals with similar midlife risk profiles into clusters and explored associations with dementia risk.

**METHODS:**

Participants without dementia at baseline (1987–1989) from the prospective Atherosclerosis Risk in Communities (ARIC) study were included (ages 45–64 years; *N *= 15,250). Using unsupervised hierarchical cluster analysis, nine clusters were created and defined based on 14 midlife morbidities. The associations with incident dementia (*N *= 3272 cases, median follow‐up 25 years) and deaths (*N *= 9099) were evaluated using time‐to‐event regression models.

**RESULTS:**

Compared with the healthiest cluster (Cluster 1), Clusters 2 (smoking) (hazard ratio [HR](95% confidence interval [CI]) = 1.62 (1.08, 2.43)), 5 (obesity, diabetes, hypertension, and hypertriglyceridemia) (HR(95%CI) = 1.91 (1.35,2.70)), and 7/8 (atrial fibrillation/heart failure) (HR(95%CI) = 2.69 (1.59,4.57)) were associated with dementia. Accounting for competing risk of death in the Fine‐Gray subdistribution model negated the cluster‐dementia association.

**DISCUSSION:**

Midlife morbidity clusters are important for dementia and mortality risk.

## Highlights

- Cluster analysis is a useful approach to capture complex morbidity patterns in the population.
- An unsupervised machine learning method yielded nine distinct clusters of morbidities in participants without dementia in the Atherosclerosis Risk in Communities (ARIC) Study cohort.
- Individuals in clusters defined by current smoking (Cluster 2), obesity, diabetes, hypertension, and hypertriglyceridemia (Cluster 5), or heart failure/atrial fibrillation (Cluster 7/8) in midlife, had a significantly increased risk of dementia compared to those in the healthiest cluster.
- These findings emphasize the importance of midlife morbidity on dementia risk in older adults, highlighting the potential for targeted early interventions based on clustered morbidity profiles.

## 1 BACKGROUND

Dementia is a major public health concern, and vascular risk factors, particularly during midlife, have been associated with the development of dementia during late‐life.^1^, ^2^, ^3^ Risk factors previously linked to dementia tend to cluster together in individuals, suggesting there may be discrete classes or subgroups of individuals with differing levels of morbidity‐associated dementia risk. Thus, it is important to consider not only the impact of the individual morbidities but also their combined effect on the late‐life outcome which can be more than the sum of the individual conditions.

In an age of personalized medicine, it is critical to understand how multiple frequently co‐occurring morbidities may modify the risk of dementia and to evaluate these combinations at an individual level. Machine learning methods, such as cluster analysis, enhance our understanding of disease co‐occurrence by identifying groups of individuals with similar clinical features and characterizing these clusters by their distinct patterns of morbidities. Prior studies utilizing cluster analysis, multimorbidity indices, and latent class analysis have consistently demonstrated the strong relationships between multimorbidity and dementia.^4^, ^5^, ^6^ This type of data‐driven analysis which focuses on shared clinical histories rather than a simple count of conditions provided novel insights into the trajectories of mortality, ischemic stroke, and dementia risk in large populations. The clinical utlility lies in identifying synergistic risk patterns: multimorbidity is not a static phenomenon but follows distinct trajectories that shape late‐life outcomes. The combined presence of certain cardiometabolic risk factors can pose a multiplicative risk of mortality that is greater than the risk of any condition alone.^4^, ^5^

This study used data from the prospective Atherosclerosis Risk in Communities (ARIC) study to assess whether clusters of morbidities in midlife previously shown to be strongly associated with ischemic stroke risk and severity were also associated with greater dementia risk.^6^ We furthermore explored whether the associations between the clusters and dementia risk were modified by demographic characteristics including sex, race, apolipoprotein E (APOE) ε4 carrier status, and level of educational attainment. Finally, we examined whether the association between midlife morbidity clusters and dementia differed based on age of dementia onset, specifically prior to 80 years of age vs. afterwards.

## 2 METHODS

### 2.1 Study population

The ARIC study is an ongoing community‐based prospective study that began in 1987–1989 investigating the risk factors and occurrence of subclinical and clinical cardiovascular disease. The study included 15,792 adults aged 45–64 years at baseline (visit 1) from four US communities (Washington County, Maryland; Forsyth County, North Carolina; Jackson, Mississippi; and suburban Minneapolis, Minnesota). Between visit 1 (year 1987–1989) and visit 8 (year 2020), participants completed 7 in‐person visits and one telephone‐based visit for visit 8 (year 2020). Dementia adjudication began at visit 5 (2011–2013), coincinding with the start of the ARIC Neurocognitive Study (ARIC‐NCS), which included detailed cognitive assessments. Additional follow‐up of ARIC study participants has been conducted through annual (semi‐annual since 2012) telephone interviews and active surveillance of hospitalizations and mortality. Detailed information about the ARIC study design has been provided elsewhere.^7^

**RESEARCH IN CONTEXT**

- **Systematic review**: Through a comprehensive literature search, we evaluated the current understanding of midlife risk factors on dementia risk. Most studies focused on individual risk factors or used composite scores of risk factor burden to assess dementia risk thereby neglecting the systemic complexity of co‐occurring morbidities in an age of personalized medicine.
- **Interpretation**: This study employed a hierarchical cluster analysis to identify clustered subpopulations sharing similar morbidity profiles in midlife. The study's findings show that those clustered subpopulations that are characterized by the accumulation of vascular and metabolic insults in midlife show an increased risk of dementia over a 33‐year period. The study also highlights the competing risk of mortality when considering dementia risk in these clustered subpopulations.
- **Future directions**: Future research should employ similar cluster algorithms in other diverse community‐based populations and explore whether the derived clusters are consistent with the study's findings. It would particularly be important to better understand how clusters of morbidities may differ in distinct populations and how these differentially relate to dementia risk.

Due to small sample size, participants who did not identify as White or Black, as well as those who were not White at the Minneapolis and Washington County sites, were excluded from the final sample (*N *= 103). Additionally, participants were excluded if they reported having had a stroke prior to baseline (*N *= 282) or had unknown indications for stroke (*N *= 3) or had date of dementia onset prior to baseline (*N *= 1). Finally, participants were excluded from these analyses if they refused to provide genetic data such as APOE ε4 genotype status, which resulted in the final sample size (*N *= 15,250; Figure S1).

The cohort study was approved by the institutional review board at each site, and written informed consent was obtained at each visit by participants, or proxies if required. This study followed the Strengthening the Reporting of Observational Studies in Epidemiology (STROBE) reporting guidelines.

### 2.2 Dementia ascertainment

Briefly, dementia diagnosis was ascertained through three approaches. First, dementia was determined based on an adjudicated diagnosis from in‐person attendance at visits 5 (2011–2013), 6 (2016–2017), or 7 (2018–2019). Several types of assessments were used including data from longitudinal cognitive and neuropsychological evaluations and informant interviews, used to generate computer‐generated algorithmic diagnoses that relied on standardized definitions of dementia which were then adjudicated by a panel of experts. Second, for those individuals who were unable to attend an in‐person visit, additional cases of dementia were identified based on predefined criteria from the Telephone Interview for Cognitive Status–Modified as well as from informant telephone interviews using a modified version of the Clinical Dementia Rating scale and the Functional Activities Questionnaire, as well as the eight‐item Interview to Differentiate Aging and Dementia (AD‐8) and six‐item screener that were used after visit 5 prospectively with informants and participants, respectively, for ongoing surveillance. Third, more dementia cases were additionally identified through surveillance data that included prior discharge hospitalization International Classification of Diseases Ninth Revision (ICD‐9) and death certificate codes for dementia. Further details outlining dementia adjudication among ARIC participants have been described previously.^1^, ^8^

### 2.3 Cluster analysis

The clusters were computed in a previous ARIC study using an agglomerative hierarchical clustering approach with k‐means consolidation (Hierarchical Clustering on Principle Components [HCPC] function, FactoMineR package),^6^, ^9^ which is an unsupervised machine learning method that does not rely on random initialization and is therefore not susceptible to variation across random seeds. Briefly, data on 14 vascular risk factors and morbidities considered to be influential in subsequent stroke risk and consistently measured during midlife and throughout subsequent ARIC visits were used to compute the clusters. Prior to cluster analysis, missing data (averaging 1.8%) was handled using multivariate imputation by chained equations method (MICE).^10^ For the purposes of this analysis, unless otherwise specified, the status of each of these morbidities was used from ARIC visit 1. Hypertension was defined by the average of the second and third of three measurements showing elevated systolic blood pressure ≥140 mm Hg, diastolic blood pressure ≥90 mm Hg, or antihypertensive medication use. Diabetes was defined by either self‐reported use of diabetic medication or insulin, a self‐reported diagnosis by a physician, having a fasting glucose level ≥126 mg/dL, or having a non‐fasting glucose level ≥200 mg/dL. Body mass index (BMI) was used to determine obesity, where participants with a BMI ≥30 kg/m^2^ were considered obese. Hypercholesteremia was defined as a total cholesterol ≥240 mg/dl and hypertriglyceridemia as total triglycerides > 177 mg/dl. Participants provided self‐reported information on current smoking, current drinking, and were asked if they had any previous cancer diagnoses. Heart failure (HF) was diagnosed using the Gothenburg criteria and documentation of current medications.^11^ Atrial fibrillation was determined using electrocardiograms conducted at baseline.^12^ The Chronic Kidney Disease Epidemiology Collaboration (CKD‐EPI) equation was used to calculate the estimated glomerular filtration rate (eGFR) by using serum creatinine level, age, race, sex, and body size. ^13^ Renal dysfunction was defined as presence of eGFR < 60 mL/min per 1.73 m^2^.^14^ Vital exhaustion (VE), as a surrogate for depression, was measured at visit 2 (not available at visit 1) using the Maastricht Questionnaire, where a score ≥13 was classified as “high VE”.^15^ Coronary heart disease (CHD) was defined as evidence of previous myocardial infarction (by adjudicated electrocardiography data at rest or by self‐reported physician diagnosis) or cardiovascular revascularization. Peripheral artery disease (PAD) was classified by a score < 0.9 on the Ankle–Brachial index (ABI) in one randomly chosen leg.

Once the machine learning algorithm grouped individuals into clusters, the observed/expected (O/E) ratio and marginal proportion of each morbidity within each cluster were used to characterize the clusters (Table 1). A morbidity was determined as being a defining feature of a cluster when having both an O/E ratio of ≥1.5 and a marginal proportion of ≥25% within that cluster. To clarify, as an example, in Cluster 6, while CHD, diabetes, and hypertriglyceridemia all had an O/E > 1.5, meaning that individuals with those conditions were at least 1.5 times more likely to be found in Cluster 6 than in the overall population, only CHD also had a marginal proportion ≥25%, meaning that at least 25% of all individuals with CHD from the overall population were allocated to Cluster 6 (Table 1), which is why CHD, as the only morbidity meeting both of these criteria, was the only defining feature for Cluster 6. The optimal number of clusters (*N *= 9) was determined based on the gain of the within‐cluster inertia.

**TABLE 1:** Defining features in morbidity clusters.

Cluster	O/E for all risk factors for which O/E ≥ 1.5	Marginal proportion ≥ 25%	Defining feature(s)^b)^
Cluster 1 (*N *= 6332)	‐	Current drinker: 45% Hypertension: 26% Hypercholesteremia: 37% Vital exhaustion: 30%	None
Cluster 2 (*N *= 2973)	Current smoker: 3.86	Current smoker: 75%	Current smoker
Cluster 3 (*N *= 719)	Cancer: 18.21	Cancer: 85%	Cancer
Cluster 4 (*N *= 560)	PAD: 26.02 HF: 1.62 Current smoker: 1.51	PAD: 95%	PAD
Cluster 5 (*N *= 3466)	Obesity: 3.15 Diabetes: 2.42 Hypertriglyceridemia: 1.71 Hypertension: 1.57	Obesity: 71% Diabetes: 55% Hypertriglyceridemia: 39% Hypertension: 35% Vital exhaustion: 27% Hypercholesteremia: 26%	Obesity, Diabetes, Hypertriglyceridemia, Hypertension
Cluster 6 (*N *= 528)	CHD: 22.10 Diabetes: 1.66 Hypertriglyceridemia: 1.63	CHD: 76%	CHD
Cluster 7 (*N *= 27)^a)^	Atrial fibrillation: 570.52 CHD: 3.27 Renal dysfunction: 3.08 Diabetes: 1.91	Atrial fibrillation: 100%	Atrial fibrillation
Cluster 8 (*N *= 615)^a)^	HF: 22.16 CHD: 3.92 Diabetes: 2.29 Hypertension: 2.26 Obesity: 1.84 Vital exhaustion: 1.82 Hypertriglyceridemia: 1.54	HF: 89%	HF
Cluster 9 (*N *= 184)	Renal dysfunction: 83.27 HF: 3.37 PAD: 3.25 Diabetes: 2.85 CHD: 2.40 Hypertension: 2.18 Hypertriglyceridemia:1.74 Vital exhaustion: 1.53	Renal dysfunction: 99%	Renal dysfunction

*Note*: Median (Q1, Q3) and N(%) unless otherwise specified.

Abbreviations: CHD, coronary heart disease; HF, heart failure; O/E, observed/expected; PAD, peripheral artery disease;

^a)^ Clusters 7 and 8 were merged in the subsequent time‐to event analysis due to Cluster 7′s small sample size.

^b)^ A defining feature was based on having both an O/E≥ 1.5 and marginal proportion≥ 25%.

### 2.4 Additional covariates

Baseline data were collected on age, sex, self‐reported race, educational attainment (less than high school, high school graduate, at least some college), and APOE ε4 allele status (APOE ε4 carrier vs. non‐APOE ε4 carrier) (TaqMan assay; Applied Biosystems, Foster City, CA). Race and center were combined as a new variable due to varying distributions of race groups across the four ARIC centers.

### 2.5 Statistical analysis

Statistical software (*R, version 4.4.1*) was used for this analysis. In a total of 662 (4.3%) participants who had consented to but had missing genetic data or were missing values for APOE ε4 carrier status or education status, these variables were imputed via MICE.^16^ Descriptive statistics were used to assess differences in demographics characteristics and dementia incidence across clusters.

The main analysis used a cause‐specific Cox proportional hazard model with years since baseline to estimate the association between midlife morbidity clusters and incident dementia, adjusting for age, sex, race‐center, APOE ε4 carrier status, and educational attainment.^17^ Participants who did not develop dementia over their respective follow‐up surveillance period were censored for analysis due to administrative censoring on December 31 2020, death prior to this date, or loss to follow‐up between baseline and 2020. The cluster without a defining morbidity, representing the healthiest profile in the cohort, was used as the reference group. Because the proportional hazards assumption was violated, as indicated by the log‐minus‐log survival plots, the follow‐up period was empirically split at 15 years based on a step function to ensure reliable hazard ratio estimates across the two distinct time intervals (baseline to 15 years, and 15 years to end of follow‐up). This specification was used in all time‐to‐event analyses. We did not adjust the alpha level for multiple comparisons because the comparisons of each cluster against the reference cluster were pre‐planned, and results were largely consistent across both intervals.

In addition to the Cox regression model in the main analysis, we also employed a competing risk model based on the Fine‐Grey method, specifically known as the sub‐distribution hazard regression model, to test whether the association between the clusters and incident dementia persisted when accounting for mortality as a competing event.^18^ While standard censoring events were handled conventionally, mortality was distinguished as the competing risk. This necessity drove the use of the sub‐distribution hazard regression model, which specifically estimates the sub‐distribution hazard ratio (sHR) to account for this competing event's influence on the cumulative incidence of dementia. In secondary analyses, interaction effects between the morbidity clusters and each covariate were evaluated in separate cause‐specific Cox proportional hazard models. Stratified analyses were then conducted by each of these demographic and genetic characteristics. We further evaluated whether the association of the clusters with incident dementia risk differed by age of dementia onset (dementia before age 80 vs. dementia at or after age 80) using a cause‐specific Cox proportional hazard model with a step function split for age of dementia onset. The model was adjusted by baseline age, sex, education, race‐center, and APOE ε4 carrier status. Visual graphical inspection of the model's proportional hazard assumption showed no evidence of violation. To better understand the association of mortality and dementia diagnosis by age 80 for each midlife cluster, an alluvial plot was created to visualize the proportion of individuals who (1) had developed dementia by age 80, (2) remained dementia‐free and were still alive by age 80, or (3) were deceased without dementia by age 80.

In a sensitivity analysis, we assessed whether the association of the clusters with dementia risk persisted among participants who did not experience an ischemic stroke event over the 33‐year follow‐up.

## 3 RESULTS

### 3.1 Defining features in the clusters

The hierarchical cluster analysis carried out in the previous study resulted in nine clusters^6^ (Table 1). The clusters were defined by the following risk factors: Cluster 1 (*N *= 6332): no defining features; Cluster 2 (*N *= 2973): current smoking; Cluster 3 (*N *= 719): cancer; Cluster 4 (*N *= 560): PAD; Cluster 5 (*N *= 3466): obesity, diabetes, hypertriglyceridemia, and hypertension; Cluster 6 (*N *= 528): CHD; Cluster 7 (*N *= 27): atrial fibrillation; Cluster 8 (*N *= 615): HF; and Cluster 9 (*N *= 184): renal dysfunction. Importantly, the defining features in many cases represent only portions of the cluster‐associated morbidity (Table 1). For instance, although not meeting both criteria as defining features, individuals in Cluster 9 also had a significantly heightened risk for HF (O/E = 3.37), PAD (O/E = 3.25), diabetes (O/E = 2.85), CHD (O/E = 2.40), and hypertension (O/E = 2.18), emphasizing the comorbid nature of these chronic diseases with renal dysfunction.

### 3.2 Cohort characteristics overall and in the clusters

Among the 15,250 participants included in this study, the median age (Q1, Q3) was 54 (49, 59) years at baseline, and approximately 55% were women (Table 2). Over 75% of the population reported having at least a high school education, and 26% self‐reported being of Black race. From baseline (1987–1989) to December 31 2020, a total of 3297 participants (21%) developed dementia.

**TABLE 2:** Demographic and other characteristics of individuals within the nine clusters.

Characteristic	Overall *N* = 15,250	Cluster 1 *N* = 6248	Cluster 2 *N* = 2951	Cluster 3 *N* = 714	Cluster 4 *N* = 554	Cluster 5 *N* = 3438	Cluster 6 *N* = 527	Cluster 7 *N* = 27	Cluster 8 *N* = 610	Cluster 9 *N* = 181
Age (years)	54 (49, 59)	53 (49, 59)	53 (48, 57)	56 (51, 60)	56 (50, 61)	54 (49, 59)	57 (52, 61)	59 (55, 63)	56 (51, 60)	60 (55, 63)
Women	8433 (55%)	3339 (53%)	1494 (51%)	475 (67%)	384 (69%)	2087 (61%)	109 (21%)	9 (33%)	439 (72%)	97 (54%)
Race‐Center										
Washington County, White	3876 (25%)	1581 (25%)	598 (20%)	199 (28%)	168 (30%)	924 (27%)	191 (36%)	9 (33%)	165 (27%)	41 (23%)
Forsyth County, Black	460 (3.0%)	117 (1.9%)	141 (4.8%)	8 (1.1%)	20 (3.6%)	106 (3.1%)	14 (2.7%)	0 (0%)	42 (6.9%)	12 (6.6%)
Forsyth County, White	3448 (23%)	1593 (25%)	796 (27%)	237 (33%)	86 (16%)	485 (14%)	116 (22%)	6 (22%)	101 (17%)	28 (15%)
Jackson, Black	3561 (23%)	1044 (17%)	725 (25%)	71 (9.9%)	154 (28%)	1233 (36%)	76 (14%)	4 (15%)	185 (30%)	69 (38%)
Minneapolis, White	3905 (26%)	1913 (31%)	691 (23%)	199 (28%)	126 (23%)	690 (20%)	130 (25%)	8 (30%)	117 (19%)	31 (17%)
Education										
Less than high school education	3599 (24%)	964 (15%)	823 (28%)	127 (18%)	181 (33%)	1035 (30%)	178 (34%)	8 (30%)	215 (35%)	68 (38%)
High school education or GED	6239 (41%)	2541 (41%)	1272 (43%)	317 (44%)	229 (41%)	1360 (40%)	198 (38%)	10 (37%)	244 (40%)	68 (38%)
At least some college	5412 (35%)	2,743 (44%)	856 (29%)	270 (38%)	144 (26%)	1,043 (30%)	151 (29%)	9 (33%)	151 (25%)	45 (25%)
APOE ε4 carrier	4516 (30%)	1782 (29%)	932 (32%)	203 (28%)	180 (32%)	1,012 (29%)	153 (29%)	2 (7.4%)	203 (33%)	49 (27%)
Dementia (overall)	3272 (21%)	1382 (22%)	525 (18%)	145 (20%)	111 (20%)	880 (26%)	76 (14%)	5 (19%)	126 (21%)	22 (12%)
Incidence of dementia per 1000 person‐years (95% CI)	9.08 (8.77, 9.40)	8.43 (7.99, 8.88)	8.09 (7.41, 8.81)	9.09 (7.67, 10.69)	9.87 (8.12, 11.89)	11.04 (10.32, 11.79)	7.97 (6.28, 9.97)	11.41 (3.70, 26.62)	10.57 (8.80, 12.58)	8.45 (5.29, 12.79)
Age of dementia onset (years)	81.42 (76.67, 85.92)	82.87 (78.36, 86.87)	79.11 (75.38, 83.50)	81.57 (76.83, 86.95)	79.97 (76.45, 85.36)	80.72 (75.87, 85.23)	81.93 (75.69, 86.27)	85.14 (80.52, 90.52)	81.13 (75.82, 85.23)	82.60 (78.85, 85.40)
Dementia before age 80	1352 (8.9%)	470 (7.5%)	282 (9.6%)	59 (8.3%)	56 (10%)	392 (11%)	29 (5.5%)	1 (3.7%)	55 (9.0%)	8 (4.4%)
Deaths (overall)	9099 (60%)	2861 (46%)	2037 (69%)	466 (65%)	405 (73%)	2230 (65%)	454 (86%)	23 (85%)	463 (76%)	160 (88%)

*Note*: Median (Q1, Q3) and N(%) unless otherwise specified.

Abbreviations: APOE, apolipoprotein E; GED, General Educational Development.

The median age per cluster increased with the morbidity burden, from age 53 in Clusters 1 and 2 to age 59 and 60 in Cluster 7 and 9, respectively (Table 2). While women were more likely to be in Cluster 3 (67%), Cluster 4 (69%), and Cluster 8 (72%), men were more likely to be found in Cluster 6 (79%) and Cluster 7 (67%). The proportions of Black participants from Jackson and Forsyth County significantly varied between the clusters, with a lower proportion in Clusters 1 and 3 and a significantly larger proportion in Clusters 8 and 9. The cumulative dementia incidence through 2020 was highest in Cluster 1 (22%), Cluster 5 (26%), and Cluster 8 (21%). Mortality between baseline through 2020 was higher in the morbid Clusters 2–9 (65%‐88%) compared to Cluster 1 (46%), and highest in Cluster 6 (86%), Cluster 7 (85%), and Cluster 9 (88%).

### 3.3 Main analysis

#### 3.3.1 Cause‐specific hazard of the clusters with dementia incidence

**FIGURE 1:**
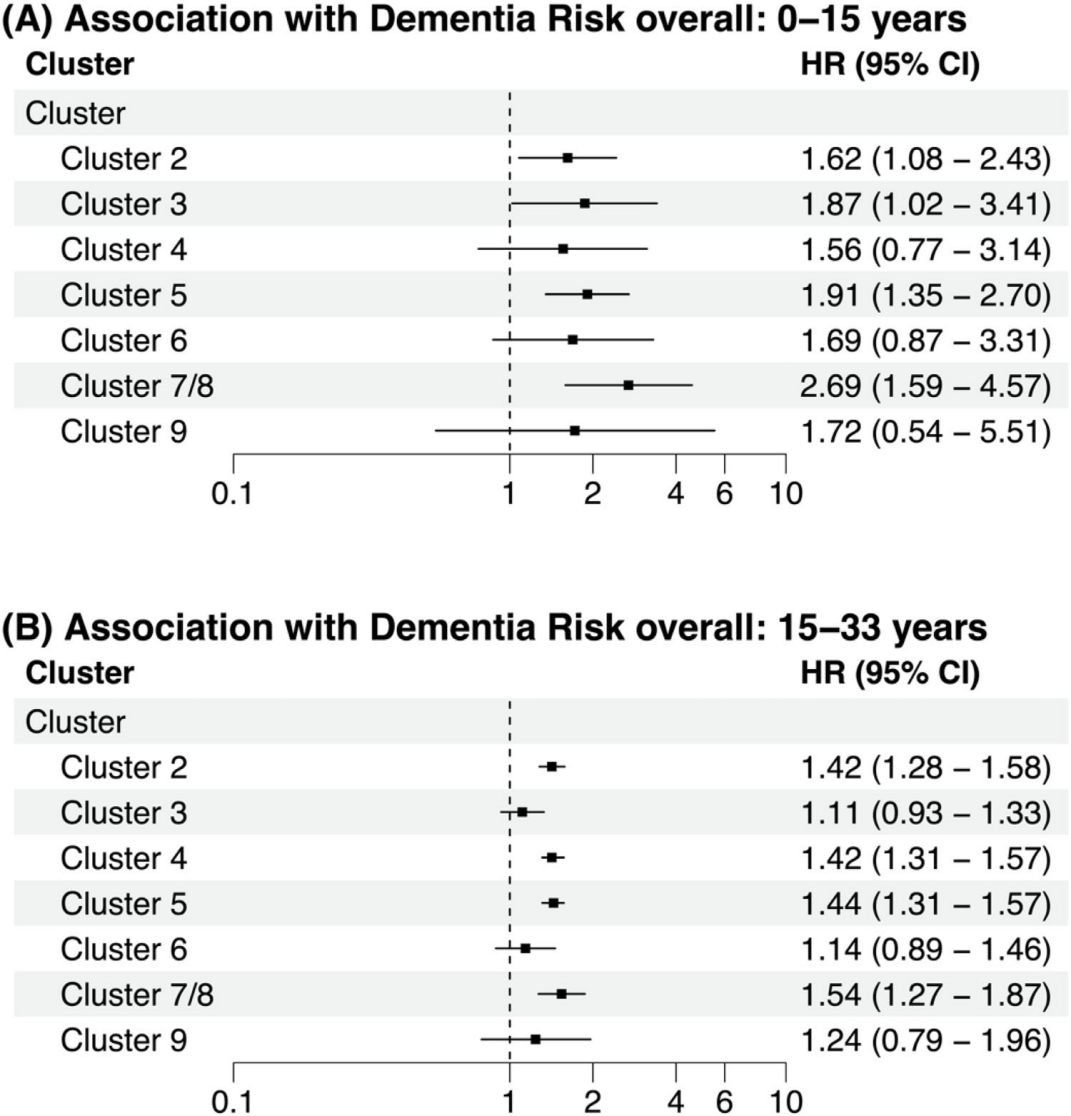
Association between the clusters defined by morbidities (Clusters 2–9) vs. healthy Cluster 1 and dementia risk. ^*^adjusted by the covariates age, sex, education, race‐center, and APOE ε4 allele status. ^†^ all HR (95% CI) refer to the association of each of the Clusters 2–9 with dementia risk when compared to Cluster 1. Cluster 1‐ no defining feature (*reference group*); Cluster 2‐ current smoking; Cluster 3‐ cancer, Cluster 4‐ PAD; Cluster 5‐ diabetes, obesity, hypertension, and hypertriglyceridemia; Cluster 6‐ CHD; Cluster 7/8‐ HF/atrial fibrillation; and Cluster 9‐ renal dysfunction. APOE, apolipoprotein E; CHD, coronary heart disease; HF, heart failure; PAD, peripheral artery disease.

Due to the small sample size of Cluster 7 (*N *= 27), Cluster 7 (atrial fibrillation) and Cluster 8 (heart failure) (*N *= 615), shown separately in Table 2, were merged into Cluster 7/8 for the time‐to‐event analysis. Cluster 1, with no defining morbidity, was used as the reference group. Participants in Cluster 2 (hazard ratio [HR](95% confidence interval [CI]) = 1.62 (1.08, 2.43)), Cluster 5 (HR(95%CI) = 1.91 (1.35, 2.70)), and Cluster 7/8 (HR(95%CI) = 2.69 (1.59, 4.57)) were at significantly higher risk of dementia compared to those in Cluster 1, when accounting for age, sex, race‐center, education, and APOE ε4 carrier status (Figure 1a–b). These associations remained consistent across both follow‐up periods, despite a significant difference in number of incident dementia cases between the two (period 1: *N *= 221; period 2: *N *= 3051). We also found that individuals in Cluster 3 (vs. Cluster 1) had a significantly higher dementia risk (HR(95%CI) = 1.87 (1.02, 3.41)) in period 1, as did individuals in Cluster 4 (vs. Cluster 1) (HR(95%CI) = 1.42 (1.31, 1.57)) in period 2.

#### 3.3.2 Competing risk of the clusters with dementia accounting for mortality

When accounting for mortality as a competing event, morbidity clusters were no longer associated with a higher dementia risk compared to Cluster 1. Instead, except for Cluster 5 (sHR(95%CI) = 1.04 (0.95, 1.13)), all morbidity clusters were significantly associated with a lower risk of dementia compared to Cluster 1(Table 3). When assigning mortality as the primary event and dementia as the competing event in the subsequent analysis, the findings showed the reverse association and all morbidity clusters had a strong positive association with mortality. These findings indicate that the absolute risk of dying without dementia in the morbidity clusters was significantly greater than the risk of experiencing dementia, which is further supported by the higher cumulative incidence function for mortality (Figure S2). To better understand the temporal relationship between dementia onset and mortality in the population, we plotted the age at which participants either developed dementia or died without dementia, in a histogram. The distribution showed that a significant proportion of the population died years before having the chance of developing dementia as a late‐life diagnosis (Figure S3).

**TABLE 3 alz71110-tbl-0003:** Competing risks sub‐distribution hazard model with dementia and mortality.

Parameter	Sub‐distribution hazard model	
	Dementia^a)^	Mortality ^b)^
Age (per 1 year)	**1.08 (1.07, 1.08)**	**1.07 (1.07, 1.08)**
Sex (ref: men)		
Women	**1.24 (1.15, 1.34)**	**0.63 (0.60, 0.67)**
Race–Center (ref: Washington County White)		
Forsyth County, Black	**0.70 (0.55, 0.89)**	**1.36 (1.17, 1.58)**
Forsyth County, White	**0.80 (0.72, 0.88)**	**1.09 (1.02, 1.17)**
Jackson, Black	**1.10 (1.00, 1.21)**	**1.23 (1.15, 1.33)**
Minneapolis, White	**0.82 (0.75, 0.91)**	1.07 (0.99, 1.15)
APOE ε4 carrier status (*ref*: neg)		
Positive	**1.71 (1.60, 1.84)**	**0.88 (0.83, 0.93)**
Education (*ref*: less than high school)		
High school, GED	**0.90 (0.82, 0.98)**	**0.86 (0.80, 0.91)**
At least some college	**0.84 (0.76, 0.92)**	**0.74 (0.69, 0.79)**
Cluster (*ref*: Cluster 1)		
Cluster 2	**0.79 (0.71, 0.87)**	**2.36 (2.21, 2.53)**
Cluster 3	**0.79 (0.67, 0.95)**	**1.88 (1.67, 2.12)**
Cluster 4	**0.70 (0.58, 0.86)**	**2.51 (2.21, 2.85)**
Cluster 5	1.04 (0.95, 1.13)	**1.66 (1.55, 1.77)**
Cluster 6	**0.52 (0.41, 0.66)**	**2.91 (2.59, 3.28)**
Cluster 7/8	**0.72 (0.60, 0.87)**	**2.57 (2.28, 2.91)**
Cluster 9	**0.35 (0.22, 0.54)**	**4.05 (3.28, 5.00)**

*Note*: Each cell contains the sub‐distribution hazard ratio and associated 95% confidence interval for the given covariate and the given hazard model.

^a)^ Mortality as competing event.

^b)^ Dementia as competing event.

Abbreviations: APOE, apolipoprotein E; GED, General Educational Development.

### 3.4 Secondary analysis

#### 3.4.1 Interactions between demographics, genetics, and clusters on dementia incidence

We observed significant effect modifications by race, sex, and APOE ε4 status in the cause‐specific Cox regression models. Analyses examining interactions between the clusters and race on dementia risk indicated significant effect modification for Cluster 7/8 (*p‐*interaction = 0.01). The stratified analysis showed that White participants (HR(95%CI) = 1.91 (1.51, 2.42)) [period 2] in Cluster 7/8 (vs. Cluster 1) were at a significantly greater risk of dementia, when compared to Black participants (HR(95%CI) = 1.06 (0.75, 1.50)) [period 2] in Cluster 7/8 (Figure S4). There was also significant effect modification by sex for Cluster 4 (*p‐*interaction = **0.01**) and Cluster 7/8 (*p*‐interaction = **0.03**). Men (HR(95%CI) = 2.66 (1.79, 3.94)) [period 2] in Cluster 4, defined by PAD, were at significantly higher risk of dementia compared to those in Cluster 1, whereas the same was not found for women in Cluster 4 (HR(95%CI) = 1.17 (0.93, 1.49)) [period 2] (Figure S5). Likewise, men (HR(95%CI) = 2.12 (1.48, 3.04)) [period 2] had a higher dementia risk in Cluster 7/8, compared to those in Cluster 1, than did women in Cluster 7/8 (HR(95%CI) = 1.35 (1.07, 1.71)) [period 2]. The sample sizes of the clusters by the effect modifiers sex and race can be found in Tables S1 and S2.There was no significant interaction effect between the clusters and education (Figure S6). A significant effect modification was, however, present by APOE ε4 carrier status for Cluster 3 and 5 (*p‐*interactions = 0.01). The stratified analysis showed that Cluster 3 (vs. Cluster 1) was associated with a significantly higher risk of dementia only among APOE ε4 carriers (HR(95%CI) = 4.28 (2.09, 8.78)) [period 1] (Figure S7), but the Clusters (3 vs. 1) did not differ in terms of dementia risk among noncarriers. Individuals who were non‐APOE ε4 carriers and were in Cluster 5 (HR(95%CI) = 1.56 (1.39, 1.74)) [period 2] had a higher dementia risk than those noncarriers in Cluster 1, compared to those who were APOE ε4 carriers, in whom the additional risk associated with being in Cluster 5 (vs. Cluster 1) was not as large (HR(95%CI) = 1.26 (1.09, 1.46)) [period 2] (Figure S7).[Table alz71110-tbl-0003]

#### 3.4.2 Association of the clusters with age of dementia onset

**FIGURE 2 alz71110-fig-0002:**
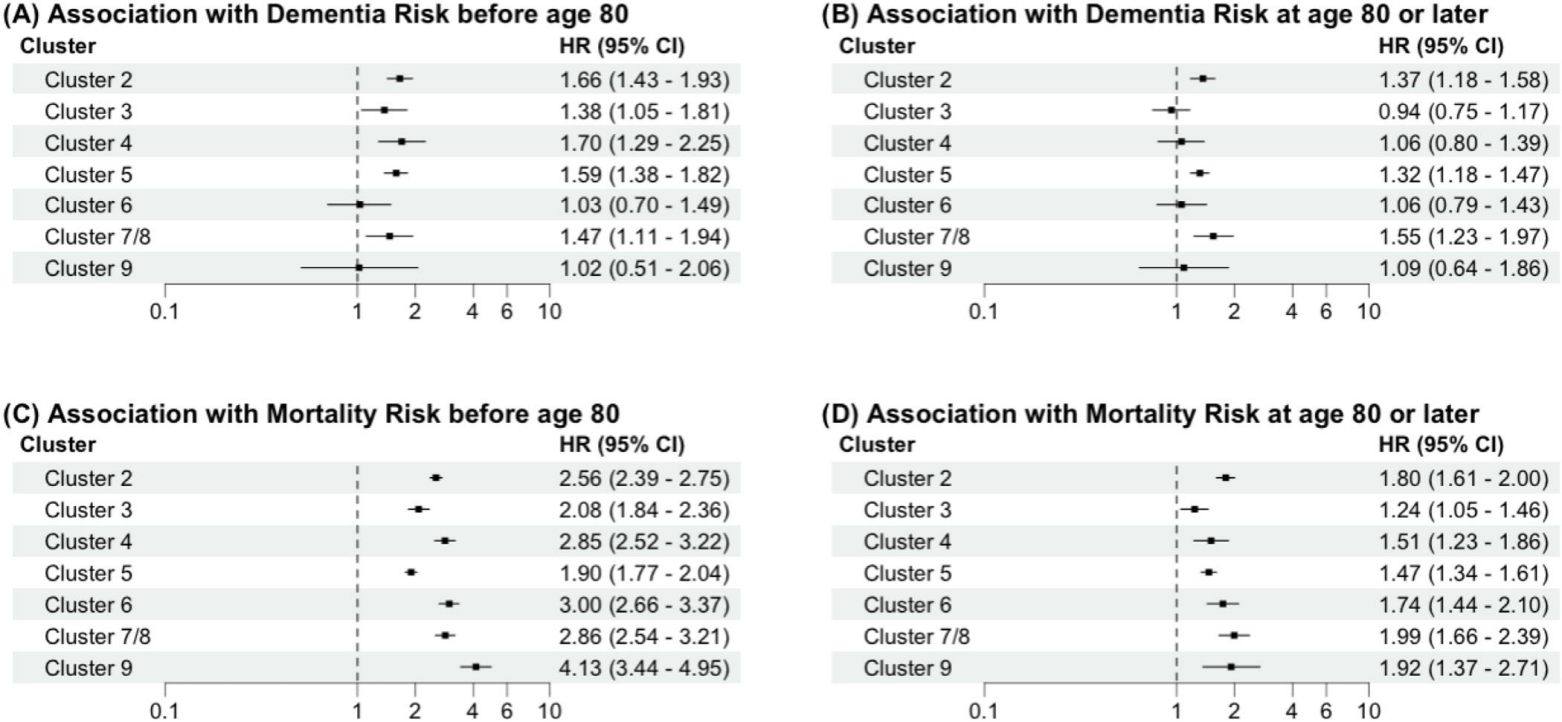
Association between the clusters defined by morbidities (Clusters 2–9) vs. healthy Cluster 1 and dementia and mortality risk, stratified by age. ^*^adjusted by the covariates age, sex, education, race‐center, and APOE ε4 carrier status. ^†^ all HR (95% CI) refer to the association of each of the Clusters 2–9 with dementia risk when compared to Cluster 1. Cluster 1‐ no defining feature (*reference group*); Cluster 2‐ current smoking; Cluster 3‐ cancer; Cluster 4‐ PAD; Cluster 5‐ diabetes, obesity, hypertension, and hypertriglyceridemia; Cluster 6‐ CHD; Cluster 7/8‐ HF/atrial fibrillation; and Cluster 9‐ renal dysfunction. APOE, apolipoprotein E; CHD, coronary heart disease; CI, confidence interval; HF, heart failure; HR, hazard ratio; PAD, peripheral artery disease.

**FIGURE 3 alz71110-fig-0003:**
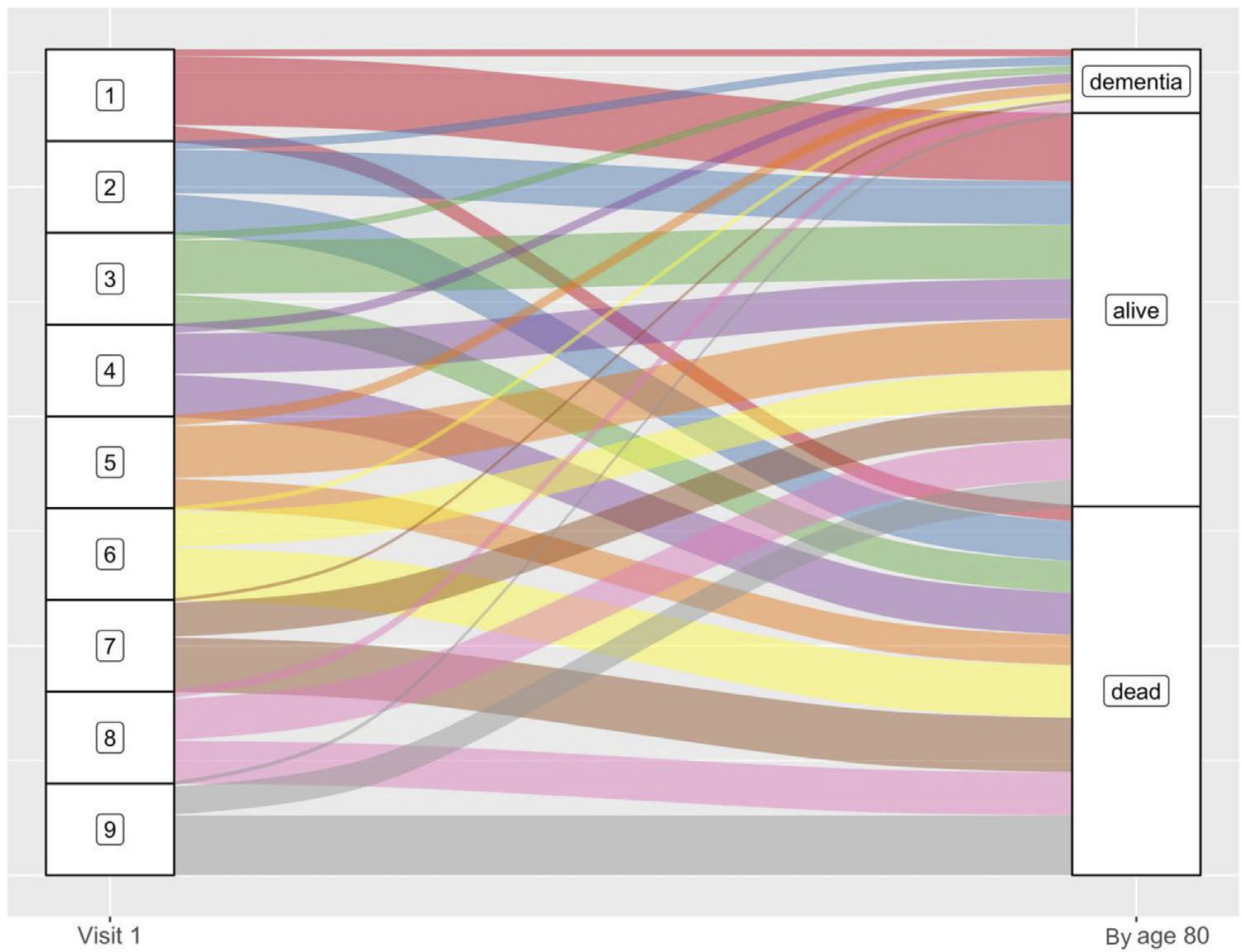
Cluster trajectories of all individuals at visit 1 based on status prior to age 80: having dementia, being alive without dementia, or being deceased without dementia.

Using a time step function with age 80 as a threshold, Cluster 2 (HR(95%CI) = 1.38 (1.05, 1.81)), Cluster 3 (HR(95%CI) = 1.38 (1.05, 1.81)), Cluster 4 (HR(95%CI) = 1.70 (1.29, 2.25)), Cluster 5 (HR(95%CI) = 1.59 (1.38, 1.82)), and Cluster 7/8 (HR(95%CI) = 1.47 (1.11, 1.94)) were associated with higher risk of dementia before age 80 (Figure 2a). The associations were similar for dementia incidence at or after age 80, with the exceptions of Cluster 3 (HR(95%CI) = 0.94 (0.75, 1.17)) and Cluster 4 (HR(95%CI) = 1.06 (0.80, 1.39)), which were no longer statistically significant (Figure 2b). When assessing the association between the clusters and mortality, all morbidity clusters were associated with a higher risk of mortality before and after age 80, when compared to Cluster 1 (Figure 2c and d). In order to gain a better understanding of the relationship between the morbidity clusters with mortality and dementia onset by age 80, an alluvial plot was created with the following categories: (1) dementia diagnosis prior to age 80, (2) alive without dementia prior to age 80, (3) deceased without dementia prior to age 80 (Figure 3). The proportion of individuals with dementia prior to age 80 was highest in Cluster 5 (11%), Cluster 2 (9.5%), and Cluster 8 (9.0%) and lowest in Cluster 6 (5.5%), Cluster 9 (4.4%), and Cluster 7 (3.7%). When it comes to mortality without dementia, the proportion of individuals who were deceased prior to age 80 increased down the morbidity hierarchy of clusters and was highest in those morbidity clusters with the lowest dementia incidence prior to age 80. More than half of the participants in Cluster 6 (61%), Cluster 7 (59%), and Cluster 9 (68%) were deceased by age 80.

### 3.5 Sensitivity analysis

#### 3.5.1 Association of the clusters with dementia in stroke‐free individuals

In the sensitivity analysis, the associations of the clusters with dementia risk did not markedly change when excluding participants who had experienced an ischemic stroke event (*N *= 1414 (9.3%)) over the 33 year follow‐up period (Figure S8). The association of Cluster 2 (HR(95%CI) = 1.62 (1.03, 2.54)), Cluster 5 (HR(95%CI) = 1.57 (1.05, 2.36)), and Cluster 7/8 (HR(95%CI) = 2.02 (1.02, 4.00)) with dementia risk remained significant, though having smaller effect sizes, when excluding individuals with incident stroke.

## 4 DISCUSSION

Our findings support a life course epidemiological framework in which midlife represents a critical period for dementia prevention. Morbidity clusters defined by current smoking (Cluster 2), diabetes, hypertension, obesity, and hypertriglyceridemia (Cluster 5) and atrial fibrillation/heart failure (Clusters 7/8) had a significantly higher risk of dementia compared to those with no defining midlife morbidities (Cluster 1), both before and after age 80. These patterns are consistent with the accumulation of vascular and metabolic insults over time that interact with neurodegenerative processes in late life. Clusters defined by cancer (Cluster 3) and PAD (Cluster 4) were not consistently associated with dementia risk across the full follow‐up period. It is conceivable that the lack of significant associations in particular for Clusters 6 and 9, defined by CHD and renal dysfunction, respectively, may be explained by the high mortality rate observed in these groups, with over half of the participants being deceased without dementia before age 80.

To test this hypothesis, we explored in greater detail the extent to which mortality influenced the observed associations between the midlife morbidity clusters and dementia risk. In contrast to the findings from the cause‐specific Cox regression model, the results from the competing risk analysis demonstrated that none of the morbidity clusters were significantly associated with a higher risk of dementia compared to Cluster 1. This is explained by the high cumulative incidence rate of mortality in the morbid clusters (Clusters 2–9). Cluster 1, which served as the reference group, was the only cluster where the risk of dying was similar to the likelihood of dementia diagnosis; consequently, these findings underscore that high hazard ratios from cause‐specific Cox models do not always translate into higher absolute dementia risk when competing mortality is considered.^19^, [Fig alz71110-fig-0002], [Fig alz71110-fig-0003]

The importance of midlife morbidities for dementia risk has been consistently demonstrated in previous studies.^1^, ^20^ Morbidities such as smoking, diabetes, and hypertension occurring in midlife significantly increase the likelihood of suffering from adverse cognitive sequelae.^1^ However, since morbidities follow distinct multimorbidity trajectories, reflecting shared risk factors, biological mechanisms, and lifestyle influences, there has been growing interest in capturing these morbidity patterns as coinciding rather than combinations of isolated conditions.^6^, ^21^ This individual‐centered approach enables the stratification of dementia risk by providing a holistic profile of a person's vascular burden, which is an immediate, actionable target for precision prevention efforts. Identifying a patient as belonging to a specific cluster, such as the metabolic–hypertensive group (Cluster 5), provides a distinct trajectory for counseling and for implementing multi‐faceted, intensive midlife interventions that address the synergistic risk of multiple co‐occurring conditions. Use of cluster analysis has become a particularly effective statistical tool for identifying these patterns, as it groups individuals together solely based on shared morbidities, capturing the nuanced relationships among coexisting diseases in the population.^6^, ^22^, ^23^

This study expands upon a previous ARIC study that examined the association between midlife clusters of morbidities and incident ischemic stroke.^6^ The consistent association of several data‐derived midlife clusters with two separate, major outcomes, that is, ischemic stroke and now dementia, lends significant support to their underlying biological coherence as distinct, high‐risk, and chronic disease patterns. Although external validation in a separate cohort is warranted, this internal consistency across outcomes supports the clusters as robust markers of vascular and neurodegenerative risk. The nuanced associations of the morbidity clusters with dementia risk highlight the complexity of the condition. Whereas a stroke event occurring between mid and late life is a more direct consequence of a single event disrupting cerebral blood flow to the brain, dementia often gradually arises in late life through several interacting pathways involving genetic predisposition combined with existing neurodegenerative and cerebrovascular diseases.^24^ Furthermore, competing risks of mortality arising from multimorbid disease states in older age may additionally explain why we did not see significant associations with dementia risk in the highly morbid midlife clusters defined by CHD and renal dysfunction.

The strength of the associations between the morbidity clusters and dementia risk varied across the two time periods (period 1: 0–15 years; period 2: 15–32 years), which we explored separately due to a lack of proportional hazards over time. Clusters defined by current smoking (Cluster 2), defined by obesity, diabetes, hypertension, and hypertriglyceridemia (Cluster 5), and defined by atrial fibrillation/heart failure (Clusters 7/8) showed a significant association across both time periods. The consistency of these findings suggests that these clusters represent particularly high‐risk midlife clusters for dementia. The association with dementia risk was strongest for individuals in Cluster 7/8, defined by atrial fibrillation and heart failure. These morbidities were, however, only the tip of the iceberg. It is important to note that the morbidity used to label a cluster is its most characteristic feature, as determined by the O/E ratio and marginal proportion criteria, but it is not its sole component. This data‐driven grouping captures the full, co‐occurring burden; in both clusters, although not meeting criteria as “defining features”, individuals were also three times more likely to have CHD and twice as likely to have diabetes as reflected in the observed/expected ratio. It is possible that the interaction of these co‐occurring morbidities within the clusters amplified the observed impact on dementia risk.^25^, ^26^, ^27^

Findings have shown that the risk of dementia increases exponentially in late life, reflecting the cumulative effects of biological, genetic, and environmental risk factors over a lifetime.^8^ Recent studies have, however, also suggested that vascular risk factors may not have the same effect on dementia risk in the oldest‐old (adults aged 90+ years).^28^ This led us to explore whether clusters of morbidities we identified in midlife posed less of a risk of dementia for individuals aged 80 and older. Our study demonstrated that all three clusters defined by the morbidities current smoking (Cluster 2); diabetes, obesity, hypertension, hypertriglyceridemia (Cluster 5); and atrial fibrillation/heart failure (Cluster 7/8) were similarly associated with dementia both before and after age 80. The findings also showed that Cluster 3 (defined by cancer) and Cluster 4 (defined by PAD) were only associated with dementia risk before age 80. The elevated risk in Cluster 3 (cancer) may be driven by mechanisms such as treatment‐related neurotoxicity (e.g., “chemobrain”) and chronic systemic inflammation, which may accelerate neurodegenerative processes.^29^ For Cluster 4 (PAD), the early risk signals advanced systemic vascular burden, where widespread atherosclerosis and chronic cerebral hypoperfusion might lead to severe small vessel disease and the accelerated onset of dementia.^30^

We also found evidence of effect modification of some of the clusters by sex, race and APOE‐4 genotype. However, these significant interactions should be interpreted with caution as they were only present in one of the two surveillance periods. Future studies may help determine whether these demographic and genetic differences in the associations between clusters and dementia risk persist in other diverse population studies.

A key strength of this study is the use of the ARIC cohort, a well‐established, community‐based population with a large representation of Black adults, allowing for the examination of racial health disparities. Furthermore, using individual‐centered cluster analysis considers the shared co‐occurrence and interaction of multiple chronic conditions, providing a better understanding of multimorbidity patterns unlike approaches that focus solely on individual risk factors.

However, the study has several limitations. Generalizability is restricted to White and Black individuals in U.S. communities similar to those in the ARIC cohort, suggesting a need for future studies with more diverse ethnic groups. The small sample size of Cluster 7 (atrial fibrillation) required merging it with Cluster 8 (heart failure), potentially diluting the cluster's specific risk association. Furthermore, due to the small subgroup sizes in some clusters, we caution against overinterpreting the significant interaction terms for sex and race. Other limitations include the fact that clusters can change over time; the inherent variability introduced by the unsupervised machine learning model; and the method used for identifying cluster‐defining features (O/E ratio and marginal proportion criteria) may not fully capture all features. Finally, reliance on administrative data for some dementia ascertainment may introduce misclassification, as might the reliance on self‐reported diagnoses for some of the risk factors and morbidities, and as an observational study, the risk of residual confounding remains.

In conclusion, this study highlights the role midlife morbidities, particularly smoking, metabolic–hypertensive conditions (obesity, diabetes, hypertriglyceridemia, and hypertension) and cardiac conditions (atrial fibrillation/HF), have on dementia risk both before and after age 80. As the population ages and the number of individuals living with dementia continues to rise, cluster‐based approaches may be informative because they effectively stratify individuals into recognizable, high‐risk phenotypes, informing precision prevention efforts that may delay dementia onset and alter long‐term morbidity trajectories. The temporal complexity of mechanisms linking midlife morbidity clusters to dementia risk necessitates future research focused on delineating the distinct contributions of chronic microvascular burden versus acute late‐life vascular events. Ultimately, this strategic approach provides opportunities for early interventions that can effectively reduce both dementia incidence and premature mortality.

## CONFLICT OF INTEREST STATEMENT

K.A.W. is an Associate editor at *Alzheimer's & Dementia*, a member of the Editorial Board of *Annals of Clinical and Translational Neurology*, and on the Board of Directors of the National Academy of Neuropsychology. All other co‐authors have nothing to disclose. This research was supported in part by the Intramural Research Program of the National Institutes of Health (NIH). The contributions of the NIH author(s) were made as part of their official duties as NIH federal employees, are in compliance with agency policy requirements, and are considered Works of the United States Government. However, the findings and conclusions presented in this paper are those of the author(s) and do not necessarily reflect the views of the NIH or the U.S. Department of Health and Human Services.

Author disclosures are available in the supporting information.

## CONSENT STATEMENT

The study was performed in accordance with the ethical standards as laid down in the 1964 Declaration of Helsinki and its later amendments or comparable ethical standards. All the Institutional Review Boards of the respective institutions approved the ARIC study and participants signed informed consent for their involvement.

## Supporting information



Supporting Information

Supporting Information
