# Supplementary material for: The influence of midlife morbidity clusters on dementia risk: The ARIC study
Source: Alzheimers Dement. 2026 Feb 9;22(2):e71110. doi: 10.1002/alz.71110 (PMC12885933; doi:10.1002/alz.71110)
Supplement: Supplementary file 2 — Supporting Information [file ALZ-22-e71110-s001.docx]

**SUPPLEMENTARY MATERIAL**

**Figure S1.** Flowchart showing the number of participants included in the study

**Figure S2.** Association between the clusters defined by morbidities (cluster 2-9) vs. healthy cluster 1 and dementia risk stratified by race

**Figure S3.** Association between the clusters defined by morbidities (cluster 2-9) vs. healthy cluster 1 and dementia risk stratified by sex

**Figure S4.** Association between the clusters defined by morbidities (cluster 2-9) vs. healthy cluster 1 and dementia risk stratified by education

**Figure S5.** Association between the clusters defined by morbidities (cluster 2-9) vs. healthy cluster 1 and dementia risk stratified by APOE ε4 carrier status

**Figure S6.** Histogram showing the number of participants developing dementia or dying by (A) years to event and (B) age of event

**Figure S7.** Cumulative Incidence function of dementia and mortality by each cluster

**Figure S8.** Association between the clusters defined by morbidities (cluster 2-9) vs. healthy cluster 1 and dementia risk in all participants and in those without incident stroke

**Table S1.** Overview about cluster size by sex

**Table S2.** Overview about cluster size by race

**Figure S1.** Flowchart showing the number of participants included in the study


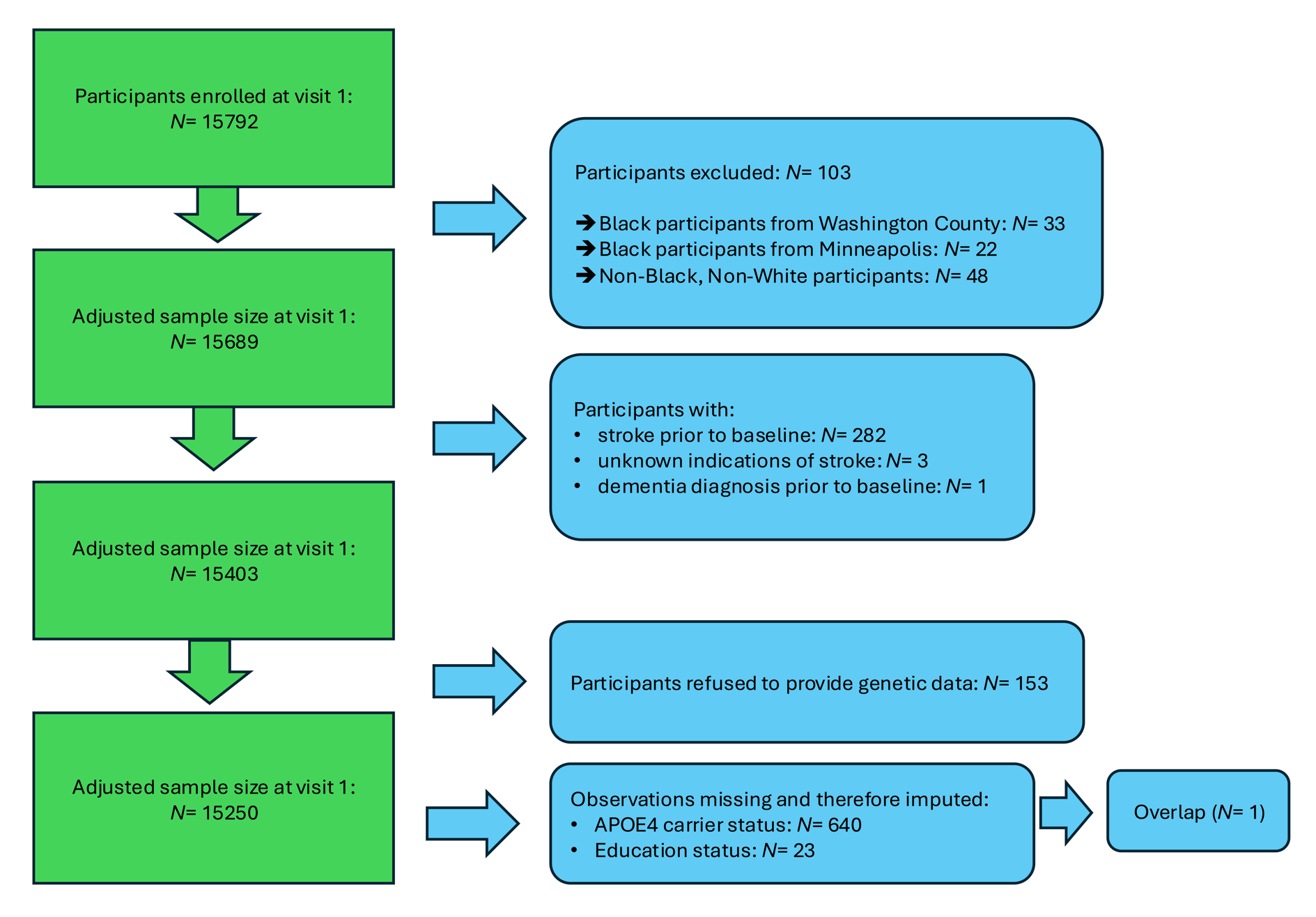


**Figure S2.** Cumulative Incidence function of dementia vs. mortality (events mutually exclusive) by each cluster

**
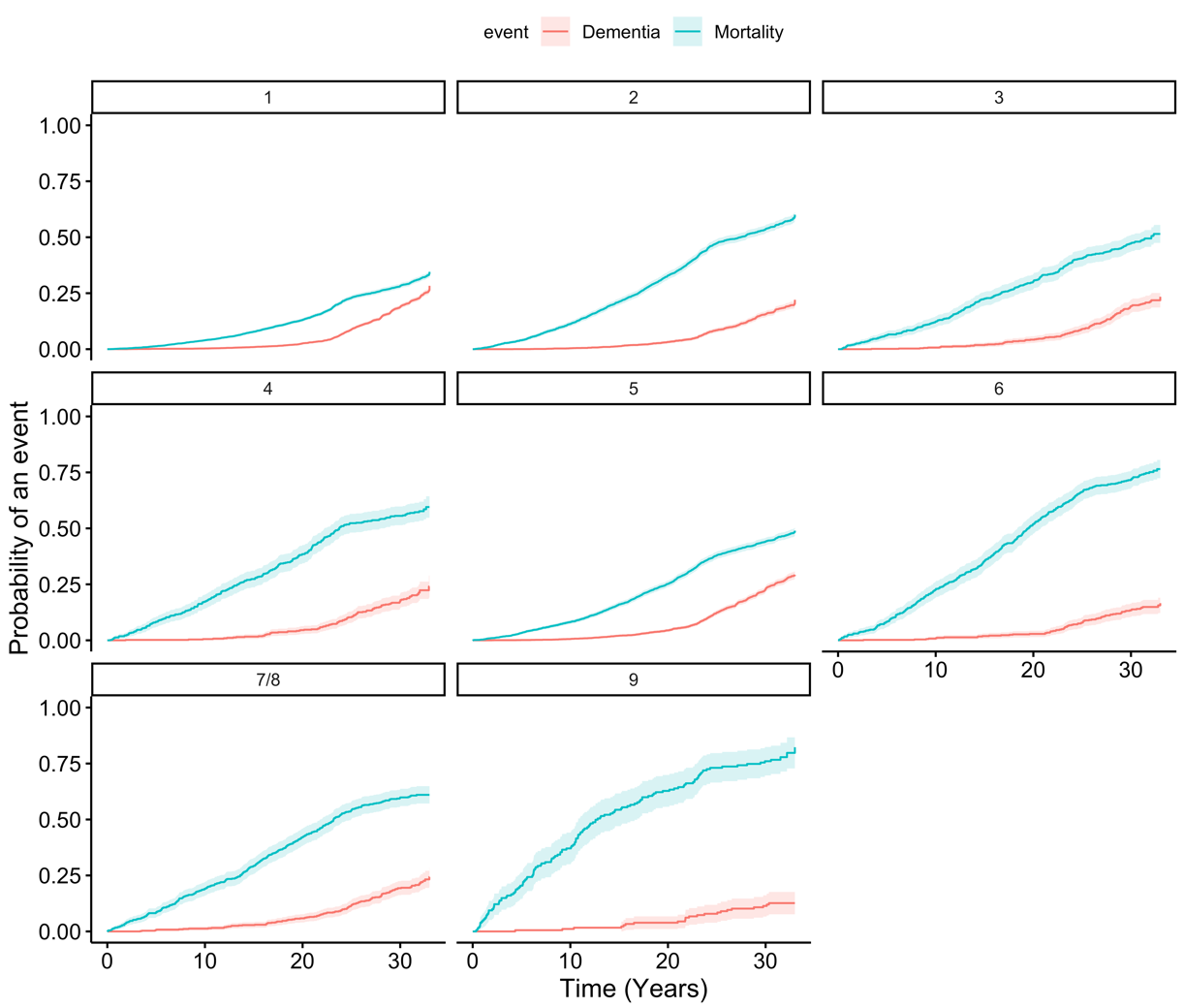
**

1. cluster 1; 2- cluster 2; 3- cluster 3; 4- cluster 4; 5- cluster 5; 6- cluster 6; 7/8- cluster 7/8; 9- cluster 9

**Figure S3.** Histogram showing the number of cases with dementia or mortality by (A) years to event and (B) age of event. The dark blue bars show the overlap in case numbers.

A)


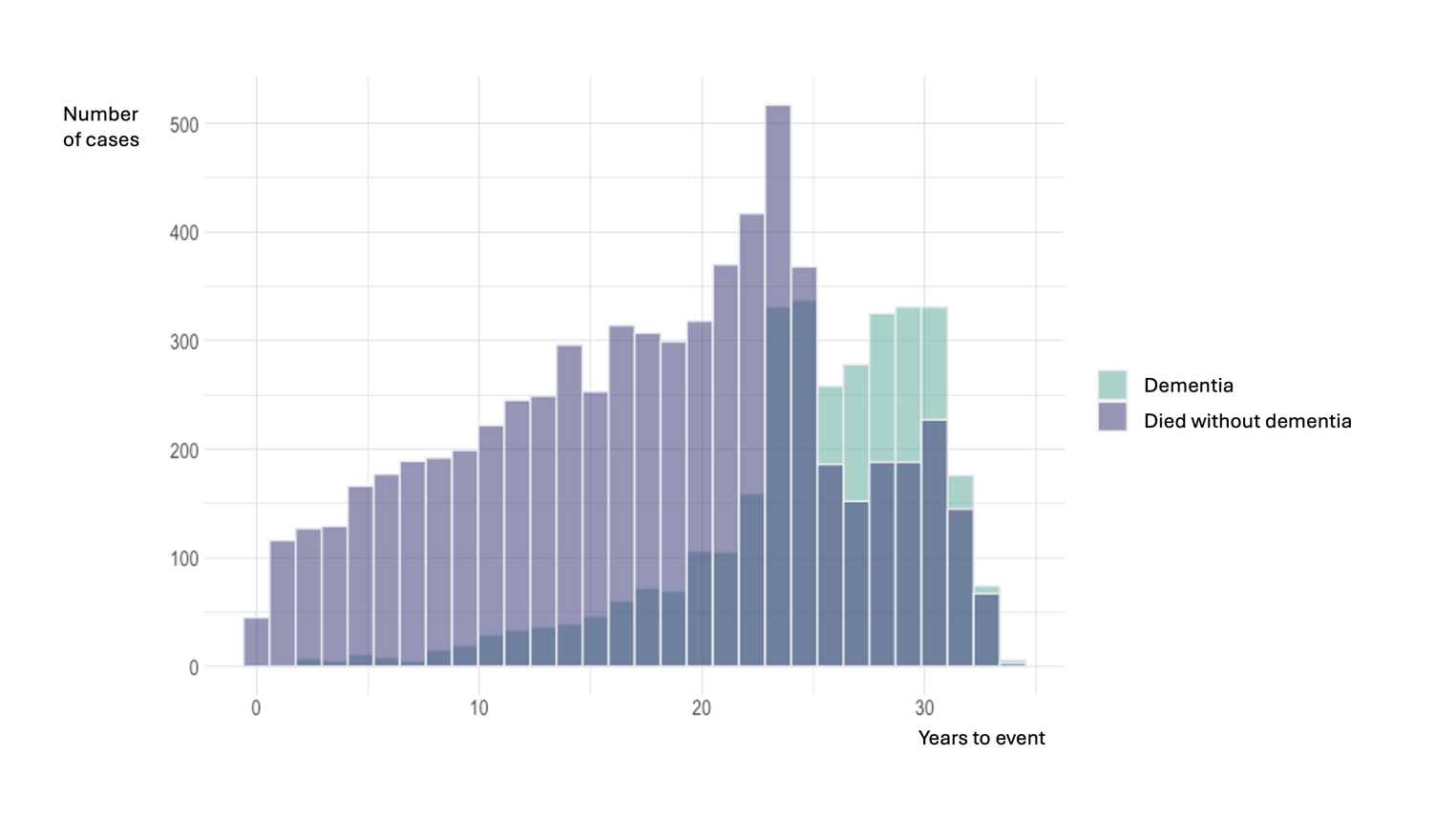


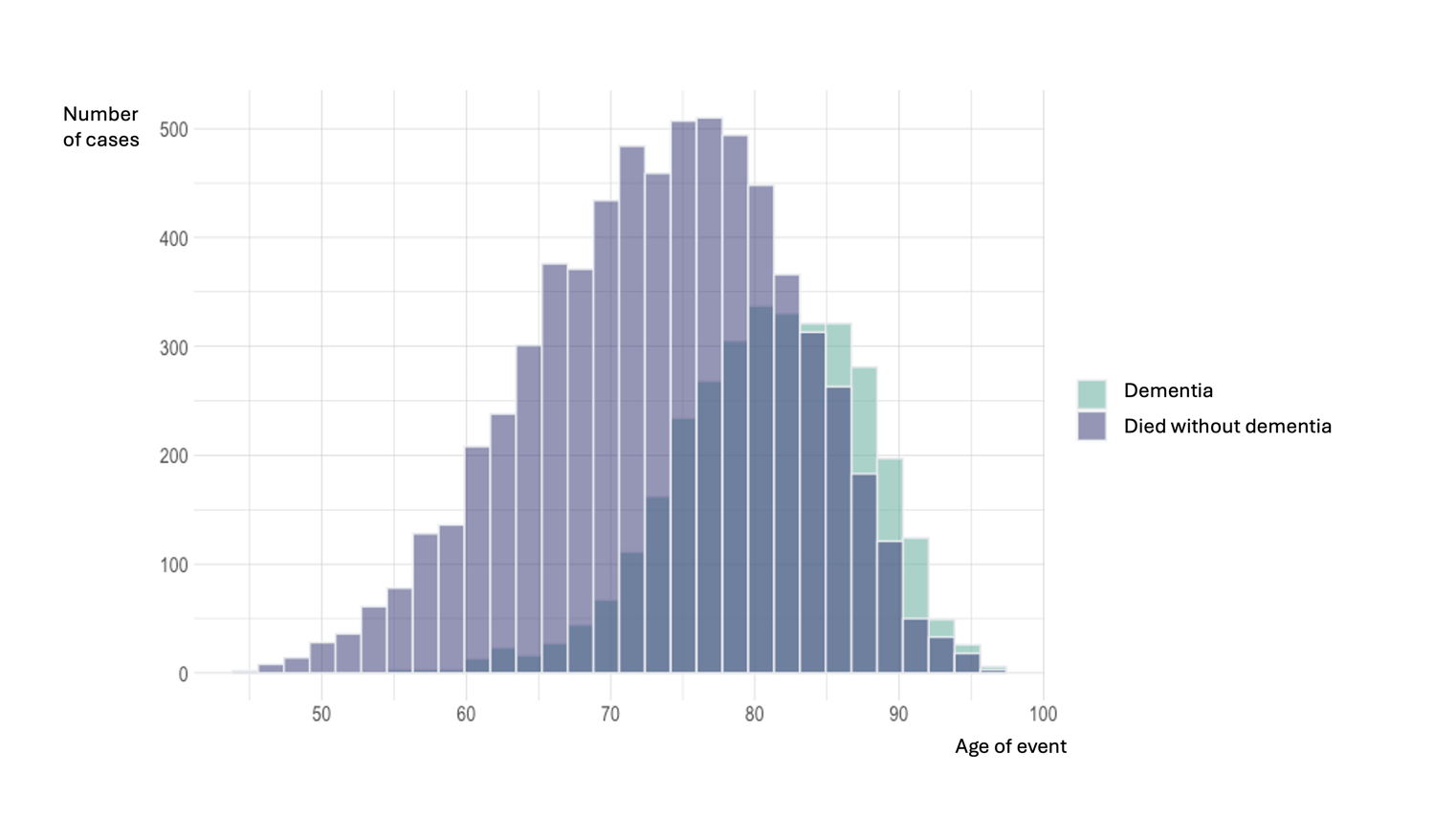
B)

**Figure S4.** Association between the clusters defined by morbidities (cluster 2-9) vs. healthy cluster 1 and dementia risk stratified by race

**
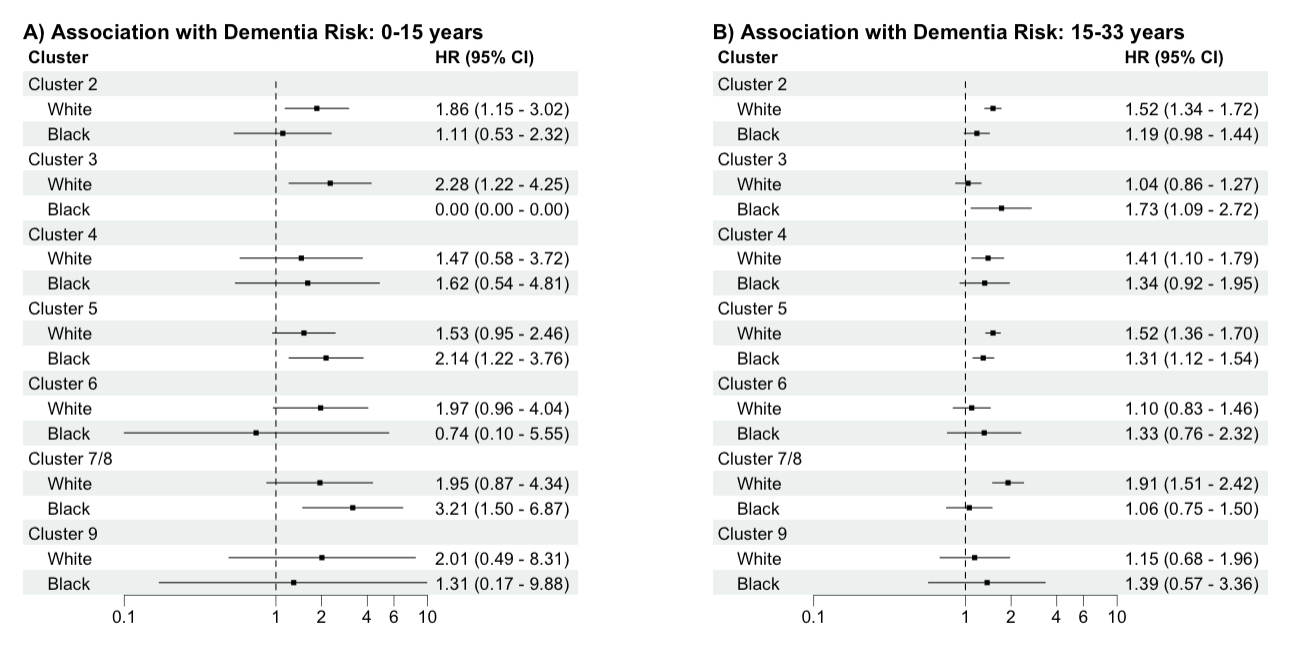
**

*adjusted by age, sex, education, and APOE ε4 carrier status

† all HR (95% CI) refer to the association of each of the clusters 2-9 with dementia risk when compared to cluster 1, within each race stratum

Cluster 1- no defining feature (*reference group*); Cluster 2- current smoking; Cluster 3- cancer, Cluster 4- PAD; Cluster 5- diabetes, obesity, hypertension, hypertriglyceridemia; Cluster 6- CHD; Cluster 7/8- HF/atrial fibrillation; Cluster 9- renal dysfunction

PAD- peripheral artery disease, CHD- coronary heart disease; HF- heart failure

**Figure S5.** Association between the clusters defined by morbidities (cluster 2-9) vs. healthy cluster 1 and dementia risk stratified by sex

**
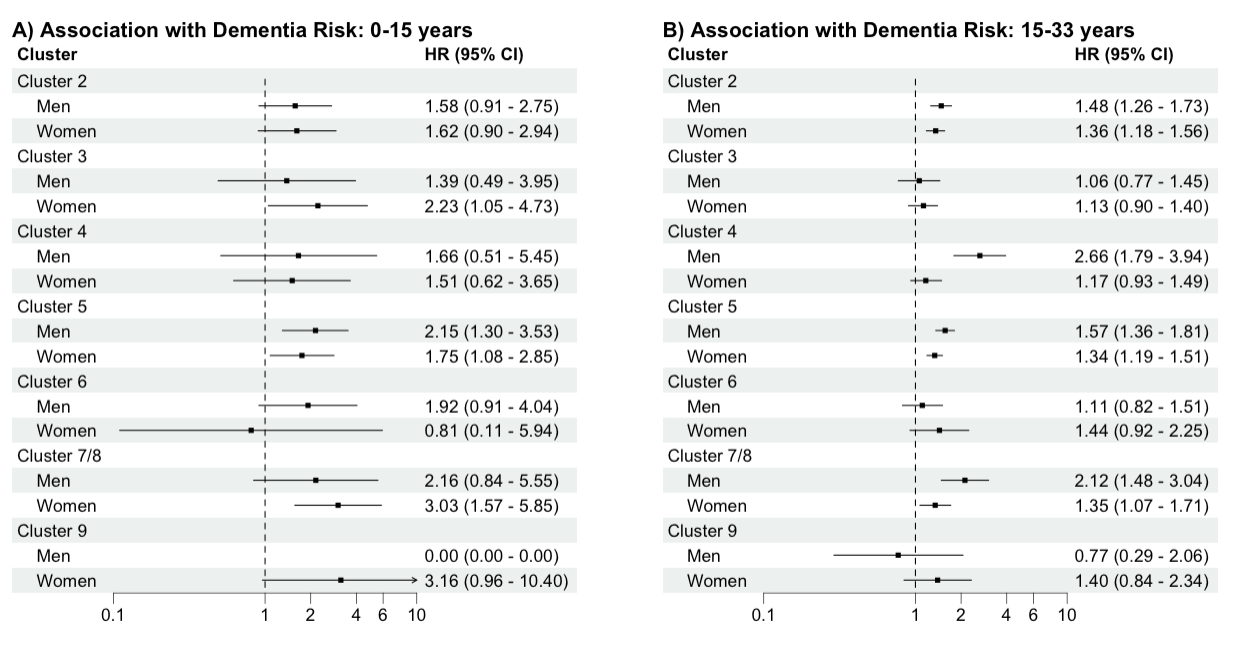
**

*adjusted by age, education, race-center, and APOE ε4 carrier status

† all HR (95% CI) refer to the association of each of the clusters 2-9 with dementia risk when compared to cluster 1, within each sex stratum

Cluster 1- no defining feature (*reference group*); Cluster 2- current smoking; Cluster 3- cancer, Cluster 4- PAD; Cluster 5- diabetes, obesity, hypertension, hypertriglyceridemia; Cluster 6- CHD; Cluster 7/8- HF/atrial fibrillation; Cluster 9- renal dysfunction

PAD- peripheral artery disease, CHD- coronary heart disease; HF- heart failure

**Figure S6.** Association between the clusters defined by morbidities (cluster 2-9) vs. healthy cluster 1 and dementia risk stratified by education

**
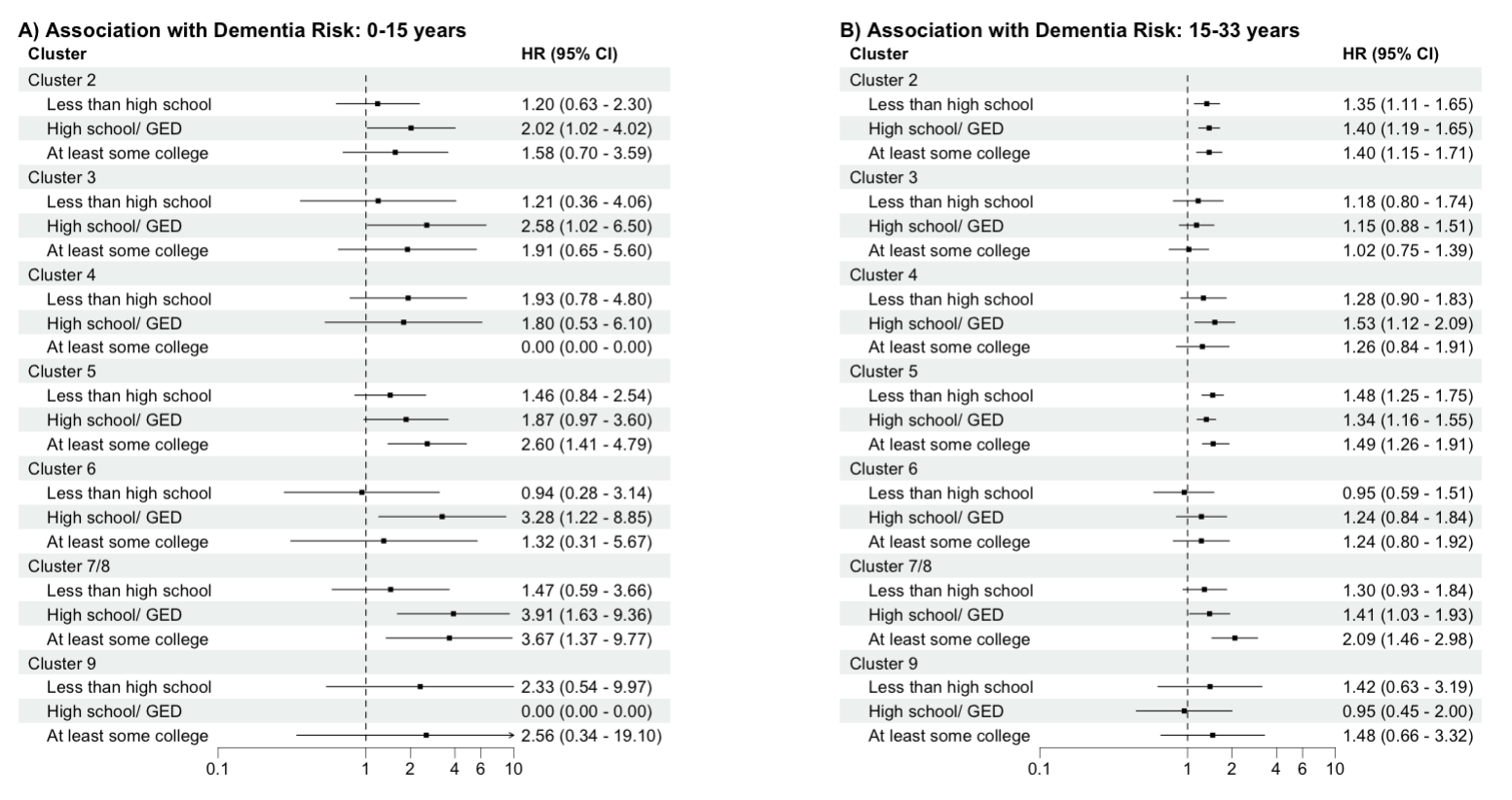
**

*adjusted by age, sex, race-center, and APOE ε4 carrier status

† all HR (95% CI) refer to the association of each of the clusters 2-9 with dementia risk when compared to cluster 1, within each education stratum

Cluster 1- no defining feature (*reference group*); Cluster 2- current smoking; Cluster 3- cancer, Cluster 4- PAD; Cluster 5- diabetes, obesity, hypertension, hypertriglyceridemia; Cluster 6- CHD; Cluster 7/8- HF/atrial fibrillation; Cluster 9- renal dysfunction

PAD- peripheral artery disease, CHD- coronary heart disease; HF- heart failure

**Figure S7.** Association between the clusters defined by morbidities (cluster 2-9) vs. healthy cluster 1 and dementia risk stratified by APOE ε4 carrier status


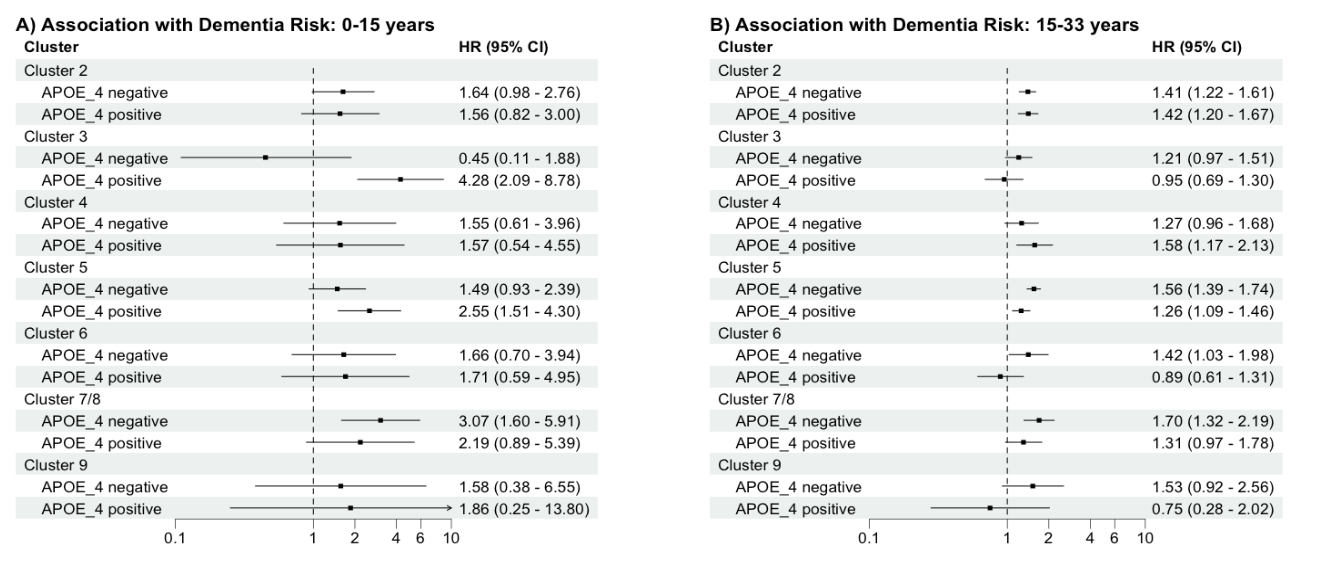


*adjusted by age, sex, education, and race-center

† all HR (95% CI) refer to the association of each of the clusters 2-9 with dementia risk when compared to cluster 1, within each APOE ε4 carrier stratum

Cluster 1- no defining feature (*reference group*); Cluster 2- current smoking; Cluster 3- cancer, Cluster 4- PAD; Cluster 5- diabetes, obesity, hypertension, hypertriglyceridemia; Cluster 6- CHD; Cluster 7/8- HF/atrial fibrillation; Cluster 9- renal dysfunction

PAD- peripheral artery disease, CHD- coronary heart disease; HF- heart failure

**
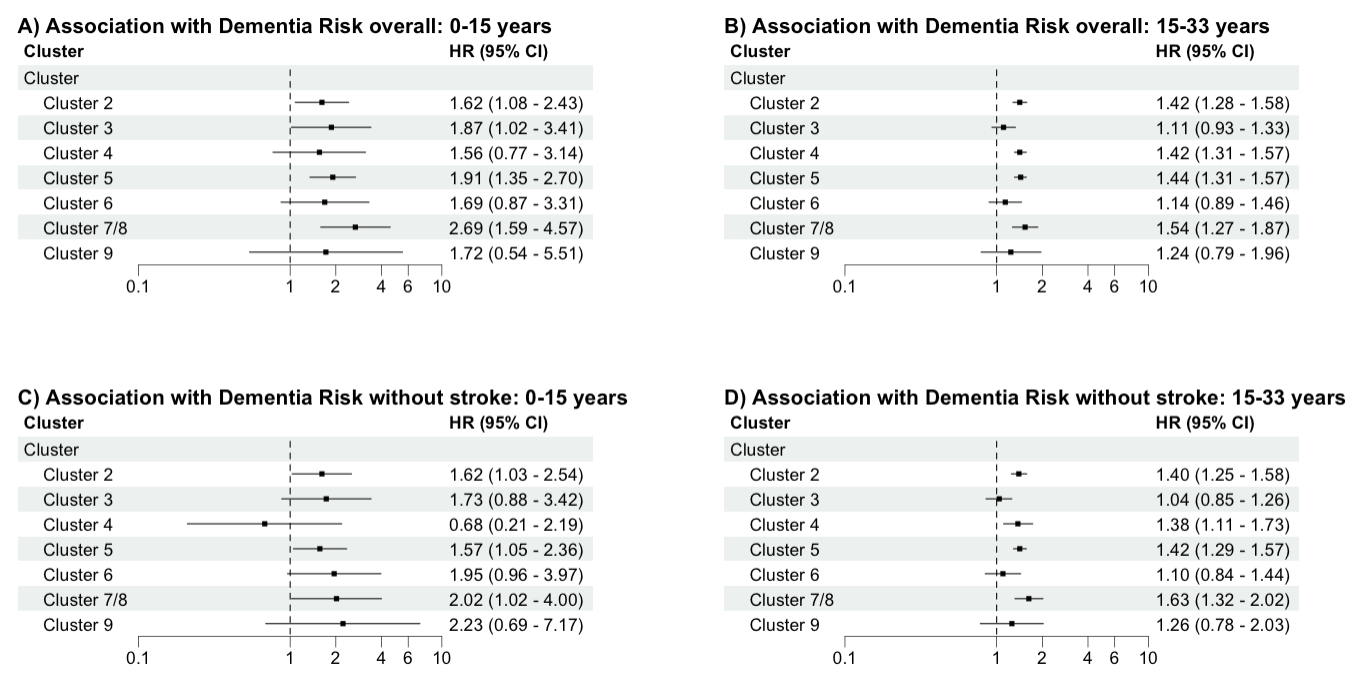
Figure S8.** Association between the clusters defined by morbidities (cluster 2-9) vs. healthy cluster 1 and dementia risk in all participants and in those without incident stroke*

*adjusted by the covariates age, sex, education, race-center, and APOE ε4 allele status

† all HR (95% CI) refer to the association of each of the clusters 2-9 with dementia risk when compared to cluster 1

Cluster 1- no defining feature (*reference group*); Cluster 2- current smoking; Cluster 3- cancer, Cluster 4- PAD; Cluster 5- diabetes, obesity, hypertension, hypertriglyceridemia; Cluster 6- CHD; Cluster 7/8- HF/atrial fibrillation; Cluster 9- renal dysfunction

*a total of 1414 participants (9.3%) experienced a stroke during the study period.

PAD- peripheral artery disease, CHD- coronary heart disease; HF- heart failure

**Table S1.** Overview about cluster size by sex

| **Cluster** | **Women (*N*)** | **Men (*N*)** |
| --- | --- | --- |
| Cluster 1 | 3339 | 2909 |
| Cluster 2 | 1494 | 1457 |
| Cluster 3 | 475 | 239 |
| Cluster 4 | 384 | 170 |
| Cluster 5 | 2087 | 1351 |
| Cluster 6 | 109 | 418 |
| Cluster 7 | 9 | 18 |
| Cluster 8 | 439 | 171 |
| Cluster 9 | 97 | 84 |

Cluster 1- no defining feature (reference group); Cluster 2- current smoking; Cluster 3- cancer, Cluster 4- PAD; Cluster 5- diabetes, obesity, hypertension, hypertriglyceridemia; Cluster 6- CHD; Cluster 7/8- HF/atrial fibrillation; Cluster 9- renal dysfunction

**Table S2.** Overview about cluster size by race

| **Cluster** | **Black (*N*)** | **White (*N*)** |
| --- | --- | --- |
| Cluster 1 | 1161 | 5087 |
| Cluster 2 | 866 | 2085 |
| Cluster 3 | 79 | 635 |
| Cluster 4 | 174 | 380 |
| Cluster 5 | 1339 | 2099 |
| Cluster 6 | 90 | 437 |
| Cluster 7 | 4 | 23 |
| Cluster 8 | 227 | 383 |
| Cluster 9 | 81 | 100 |

Cluster 1- no defining feature (reference group); Cluster 2- current smoking; Cluster 3- cancer, Cluster 4- PAD; Cluster 5- diabetes, obesity, hypertension, hypertriglyceridemia; Cluster 6- CHD; Cluster 7/8- HF/atrial fibrillation; Cluster 9- renal dysfunction
